# Establishment and characterization of a human intrahepatic cholangiocarcinoma cell line derived from an Italian patient

**DOI:** 10.1007/s13277-015-4215-3

**Published:** 2015-10-20

**Authors:** Giuliana Cavalloni, Caterina Peraldo-Neia, Chiara Varamo, Laura Casorzo, Carmine Dell’Aglio, Paola Bernabei, Giovanna Chiorino, Massimo Aglietta, Francesco Leone

**Affiliations:** 1 Medical Oncology Division, Fondazione del Piemonte per l’Oncologia (FPO), Candiolo Cancer Institute IRCCS, Strada Provinciale 142, Km 3,95, 10060 Candiolo, Turin Italy; 2 Department of Oncology, Candiolo Cancer Institute IRCCS, University of Turin, Strada Provinciale 142, Km 3,95, 10060 Candiolo, Turin Italy; 3 Unit of Pathology FPO, Candiolo Cancer Institute IRCCS, Strada Provinciale 142, Km 3,95, 10060 Candiolo, Turin Italy; 4 Flow Cytometry Center, (FPO), Candiolo Cancer Institute IRCCS, Strada Provinciale 142, Km 3,95, 10060 Candiolo, Turin Italy; 5 Cancer Genomics Laboratory, Fondazione Edo ed Elvo Tempia Valenta, Biella, Italy

**Keywords:** Intrahepatic cholangiocarcinoma, New cell line, In vitro model

## Abstract

Biliary tract carcinoma is a rare malignancy with multiple causes, which underlie the different genetic and molecular profiles. Cancer cell lines are affordable models, reflecting the characteristics of the tumor of origin. They represent useful tools to identify molecular targets for treatment. Here, we established and characterized from biological, molecular, and genetic point of view, an Italian intrahepatic cholangiocarcinoma cell line (ICC), the MT-CHC01. MT-CHC01 cells were isolated from a tumor-derived xenograft. Immunophenotypical characterization was evaluated both at early and after stabilization passages. In vitro biological, genetic, and molecular features were also investigated. In vivo tumorigenicity was assessed in NOD/SCID mice. MT-CHC01cells retain epithelial cell markers, EPCAM, CK7, and CK19, and some stemness and pluripotency markers, i.e., SOX2, Nanog, CD49f/integrin-α6, CD24, PDX1, FOXA2, and CD133. They grow as a monolayer, with a population double time of about 40 h; they show a low migration and invasion potential. In low attachment conditions, they are able to form spheres and to growth in anchorage-independent manner. After subcutaneous injection, they retain in vivo tumorigenicity; the expression of biliary markers as CA19-9 and CEA were maintained from primary tumor. The karyotype is highly complex, with a hypotriploid to hypertriploid modal number (3n+/−) (52 to 77 chromosomes); low level of HER2 gene amplification, TP53 deletion, gain of AURKA were identified; K-RAS G12D mutation were maintained from primary tumor to MT-CHC01 cells. We established the first ICC cell line derived from an Italian patient. It will help to study either the biology of this tumor or to test drugs both in vitro and in vivo.

**Electronic supplementary material:**

The online version of this article (doi:10.1007/s13277-015-4215-3) contains supplementary material, which is available to authorized users.

## Introduction

Biliary tract carcinoma (BTC) is a malignant neoplasm derived from cholangiocytes in different tracts of biliary tree. Recent studies suggest that BTC could originate from a combination of cholangiocytes, peribiliary gland around bile duct, and progenitor of hepatocytes or hepatocytes. It can be classified into intrahepatic cholangiocarcinoma (ICC), originating from the bile ducts within the liver, and extrahepatic cholangiocarcinoma (ECC) arising from bile duct outside the liver; ECC can be further classified as perihilar and distal BTC [1].

Incidence and mortality rates from BTC, including ICC (10 % of primary liver cancers in Western countries), are increasing worldwide [2–5]. Patients with unresectable disease (70–90 %) have a poor prognosis with a survival of less than 12 months following diagnosis. Conventional chemotherapy, gemcitabine alone or in association with platinum derivatives, and radiotherapy have not shown to be effective in improving long-term survival [6, 7].

The lack of effective therapies against this tumor prompts to investigate its molecular pathogenesis. Literature data extensively described risk factor promoting BTC; in particular, the etiology is different according to geography and ethnicity: infestation of the liver flukes, *Opisthorchis viverrini*, and *Clonorchis sinensis* are common in Thailand, Vietnam and Laos [8]; hepatolithiasis in Asian countries; and primary sclerosing cholangitis (PSC) in the Western countries [9]. Other potential risk factors, which affect almost all the countries, include cirrhosis, hepatitis B (HBV), hepatitis C viral (HCV), and HIV infections, inflammatory bowel disease independent of PSC, alcohol, smoking, fatty liver disease, cholelithiasis, and choledocholithiasis [10–12]. Altogether, these factors contribute to the development of the BTC and they are on the basis of genetic (gene mutations, chromosomal aberrations) and molecular (transcriptional profiles, aberrant signaling pathways) variability of these tumors [2].

To better characterize BTC, cancer cell lines represent an important and indispensable tool. By 1980s, a number of BTC cell lines, in particular originating from ECC, has been established and reported in the literature, from Japanese, Korean, Thai, or Chinese patients [13–32]. Only a limited number of ICC cell lines has been described in literature. In particular, to date, no human ICC cell line has been established from a Western countries’ patient.

In this work, we describe a human Italian ICC cell line obtained from a patient-derived xenograft (PDX) [33]. This cell line could provide a new suitable model for preclinical studies of molecular pathogenesis or drug efficacy.

## Materials and methods

### Establishment of ICC cell line from a PDX

The PDX was obtained from a tumor sample of a 60-year-old Italian woman who underwent surgical resection for ICC. Biological material was obtained from patient who has signed the informed consent, following institutional review board-approved protocols (“PROFILING Protocol, no. 001-IRCC-00 IIS-10” approved by Comitato Etico Interaziendale of A.O.U. San Luigi Gonzaga, Orbassano, Torino, Italy). This institutional study provides molecular genetic analysis, set up of primary cultures and the creation of PDX from tumor biological samples (primary tumor, metastasis, tumor cells taken under paracentesis or thoracentesis procedures, and blood). The tumor was histopathologically classified as pT2b pN0, moderately differentiated (G2) intrahepatic bile duct carcinoma. Tumor sample was also evaluated for the presence of HBV or HCV markers, resulting negative. The primitive tumor was associated with chronic cholecystitis, but not associated with liver cirrhosis or chronic liver disease, primary sclerosing cholangitis, diabetes, obesity, alcohol consumption, and tobacco smoking.

For PDX establishment, non-obese diabetic (NOD)/Shi-severe combined immunodeficient (SCID) female mice (4–6 weeks old) (Charles River Laboratory) were maintained under sterile conditions in micro-isolator cages at the animal facilities of the IRCCS-Candiolo. All animal procedures were approved by the Institutional Ethical Committee for Animal Experimentation (Fondazione Piemontese per la Ricerca sul Cancro) and by the Italian Ministry of Health. Mice were subcutaneously (s.c.) grafted with a fragment of 4 × 4 mm of representative primary tumor. After 4 months, the primary tumor was successfully engrafted in mice at first generation and was named CHC001 PDX; after reaching a volume of about 200 mm^3^, tumor was explanted and re-implanted in new mice for a second generation. The stabilization was obtained in fourth generation. To obtain the cell line, tumor specimen derived from fourth generation of PDX, was enzymatically digested with collagenase (200 U/ml) (Sigma-Aldrich, St. Louis, MO, USA) for 3 h at 37 °C. Collagenase was inactivated by two washes in fetal bovine serum (FBS) (all from Sigma-Aldrich, St. Louis, MO, USA). Single-cell suspension was obtained by filtering the supernatant through a 70-μm cell strainer (BD Biosciences, San Jose, CA). Cells were finally re-suspended in three different culture conditions: complete DMEM, RPMI, and Knockout/DMEM/F-12 media at a cell density of 300,000/mL in six-well tissue culture plates at 37 °C in a humidified atmosphere of 95 % air and 5 % CO_2_. Media were replaced twice a week. When cells reached 70–80 % of confluence, they were propagated in the optimal culture condition (complete Knockout/DMEM/F-12 medium). The stable cell line was named MT-CHC01.

### Cell lines

The ICC cell line HuH28 (Cell Bank, RIKEN Bioresource Center Riken Cell Bank, Japan) was cultured in RPMI 1640 containing 10 % FBS (all from Sigma-Aldrich, St. Louis, MO, USA), 100 U/mL penicillin and 100 μg/mL streptomycin (P/S Life Technologies Gathersburg, MD). The extrahepatic cholangiocarcinoma (ECC) WITT cells (provided by Dr. Andersen, BRIC Center, Copenhagen, Denmark) were cultured in DMEM (Sigma-Aldrich) 10 % FBS. The authentication of all the cell lines was performed by using Cell_ID system (Promega) comparing their profile with those published on the DMSZ database.

### Flow cytometry analysis

The immunophenotype of MT-CHC01 cells was determined by flow cytometric analysis. Cells were washed in 1× PBS containing 0.1 % bovine serum albumin (BSA, Sigma-Aldrich, Saint Louis, MO, USA) and 0.01 % sodium-azide. For cell permeabilization, when requested, the Fix and Perm reagent (BD Italia) was used following the manufacturer’s instructions. The following antibodies were used: fluorescein isothiocyanate (FITC)-conjugated mouse anti-CK7 and anti-CK19 (Abcam Cambridge, UK) and CD44 (BD Bioscience Europe), allophycocyanin (APC)-conjugated mouse mAbs anti-EPCAM (BD), anti-CD34 and anti-CD133 (Miltenyi BiotecS.r.l., Italy), phycoerytrin-conjugated (PE) anti-CD24, anti-CXCR4, anti-Oct3/4, anti-FOXA1/2, anti-PDX1 (all from BD), and anti-CD338 (R&D Systems, Inc. Minneapolis, MN), PerCP-Cy 5.5-conjugated anti-SOX2/17, Alexafluor 647-conjugated anti-Nanog and anti-Stro1 (BD), and Alexafluor 488-conjugated anti-PAX6 (BD).

### Mycoplasma detection

The presence of mycoplasma DNA was tested by the PCR kit VenorGeM (Minerva Biolabs) following the manufacturer’s instructions. The primers are specific to the highly conserved 16S rRNA coding region in the mycoplasma genome. Detection requires 1–5 fg of mycoplasma DNA.

### Population doubling time

The population doubling time at passage 25 was determined in hemacytometer chamber by staining with trypan-blue dye. Briefly, 1.4 × 10^5^ cells were plated in 24-well plates in triplicate in optimal medium. Viable cells were counted at 24, 48, and 72 h after seeding. The average number of cells was calculated in three different experiments.

To calculate the population doubling time (DT), we used the following formula: DT = T ln2 / ln(Xe/Xb), in which T is the time duration of culture, Xe is the cell number at the end of the incubation time and Xb is the cell number at the beginning of the incubation time.

### Anchorage-independent growth assay

Anchorage-independent growth was assessed by colony-formation assay in soft agar culture. Briefly, 100,000 cells were suspended in 0.5 ml 0.3 % (*w/v*) soft agar layered over 0.3 % (*w/v*) base agar in 24-well plates in quadruplicate. Complete medium was added twice a week for the entire period of incubation (3 weeks).

### Sphere-formation assay

MT-CHC01 cells were seeded onto six-well plates (ultra-low attachment surface) at 1.5 × 10^5^ cells per well in stem cell medium serum free (SC medium: DMEM-F12 medium, 1× B27, 200 ng/mL human EGF, 10 ng/mL human FGF, 0.4 % BSA, 4 μg/mL insulin and P/S). Sphere formation was monitored on days 7, 10, and 14 after seeding.

### In vitro motility

Motility was performed by wound-healing assay and by 8.0-μm transwell chambers (Costar, Cambridge, MA, USA). For wound-healing assay, cells were seeded in triplicate in six-well tissue culture plates and allowed to grow until 100 % confluence. The cell layer was gently “wounded.” Cell migration toward the scraped area was observed in nine randomly selected microscopic fields for each time point (up to 72 h). Images were acquired with a Leica DM13000B Inverted Microscope (Leica). The gap distance was analyzed using ImageJ software; 0 % represents the time of wound (T0). Wound closure was calculated in three different experiments. The percentage of closure was calculated as 100 − (T/T0) × 100, where T represents the average wound closures at the different time points. Statistical analysis was performed using one-way ANOVA and multiple comparison test (GraphPad software). The assay was performed in three different experiments.

Motility was also performed by transwell chambers assay (1 cm^2^/well, BD Falcon). The upper and lower cultures were separated by an 8-μm pore size poly-vinyl-pyrrolidone-free polycarbonate filters (BD Falcon). The experiments were carried out in triplicates.

After the incubation period, filters were fixed with methanol and stained with 0.5 % crystal violet in 25 % methanol; cells on the upper surface of the filters were removed using cotton swabs. Cells invading the lower surface were counted in five random fields and expressed as number of invading cells per well.

### Tumorigenicity in NOD/SCID Mice

MT-CHC01 cells at passage 35 were grown to 80 % confluence and trypsinized. For in vivo studies, NOD/Shi-SCID female mice (4–6 weeks old) of about 20–25 g/each (Charles River Laboratory) were maintained under sterile conditions in micro-isolator cages at the animal facilities of the IRCCS-Candiolo. All animal procedures were approved by the Institutional Ethical Committee for Animal Experimentation (Fondazione Piemontese per la Ricerca sul Cancro) and by the Italian Ministry of Health.

In three independent experiments, ten mice were subcutaneously (s.c.) injected into the right flank under anesthesia (mixture of isoflurane and nitrous oxide) with 3.0 × 10^6^ MT-CHC01 cells in 50 % growth factor-reduced BD Matrigel (BD Biosciences, San Jose, CA). Tumor diameters were measured weekly after cell injection up to tumor engraftment until 35 days after injection.

### Immunohistochemistry

The expression of CA19-9, alpha fetoprotein (AFP), and carcinoembryonic antigen (CEA) was evaluated. Tissues derived from primary tumor, PDX, MT-CHC01 cell line, and its xenograft were fixed in 10 % buffered formalin and embedded in paraffin. The sections (4 μm thick) were incubated in 0.01 mol/L citrate solution (pH 6) for 5 min three times at intervals of 20 min; then, the sections were soaked in 3 % H_2_O_2_ to block endogenous peroxidase activity, followed by washing in PBS at pH 7.4. The sections were incubated with the primary antibodies for AFP, CEA, CA19-9 (all from DAKO Corporation) for 30 min. Rabbit anti-human polyclonal antibody was used for AFP and CEA, and mouse anti-human monoclonal antibody was used for CA19-9. Subsequently, sections were incubated with biotinylated antibody and peroxidase-labeled streptavidin. Staining was completed after incubation with the freshly prepared substrate–chromogen solution of 3,3′-diaminobenzidine (DAB). Finally, sections were counterstained with Meyer’s hematoxylin.

### Chromosome analysis

Established cell line was subjected to chromosomal analysis using G banding and Multi-Color FISH (M-FISH). Cells were trypsinized using trypsin/EDTA after colcemid treatment (10 μg/ml) for 1 to 3 h, and slides were prepared according to standard methods. Briefly, cells were incubated in 0.075 M KCl hypotonic solution for 20 min and fixed in methanol-glacial acetic acid (3:1). G banding was performed using 2xSSC at 68 °C for 2 min and Wright’s stain for 2 min. Metaphase images were captured using an Olympus BX61 microscope (Olympus Corporation, Tokyo, Japan) and analyzed by CytoVision software (Leica Biosystems, Newcastle Ltd, UK). An average banding resolution of 300 bands was achieved. Aberrations were described according to the International System for Human Cytogenetic Nomenclature, 2013 [34].

M-FISH was performed with the aim of identifying complex chromosomal rearrangements. The probe cocktail containing 24 differentially labeled chromosome-specific painting probes (24xCyte kit MetaSystems, Altlussheim, Germany) was denatured and hybridized to denatured tumor metaphase chromosomes according to the manufacturer’s protocol for the Human Multicolor FISH kit (MetaSystems). Briefly, slides were incubated at 70 °C in saline solution (2xSSC), denatured in NaOH, dehydrated in ethanol series, air-dried, covered with 10 μl of probe cocktail (denatured) and hybridized for 2 days at 37 °C. The slides were then washed with post-hybridization buffers, dehydrated in ethanol series, and counter-stained with 10 μl of DAPI/antifade. The signal detection and analysis of subsequent metaphases used the Metafer system and Metasytems’ ISIS software (software for spectral karyotypes).

### Comparative genomic hybridization array

Genomic DNA from MT-CHC01 was extracted by Qiamp DNA mini Kit (Qiagen), and high-resolution oligonucleotide comparative genomic hybridization array (CGH) was performed following standard operating procedures from Agilent Technologies. One thousand nanograms of DNA were digested by a double enzymatic digestion (AluIþRsa I), fragmented, amplified, and purified. After the quantification with NanoDrop, 2 μg of genomic DNA of both tumor DNA and control DNA from Promega (Human Genomic DNA Female N 30742202/male N 30993901) were labeled with CY5-dCTPs and CY3-dCTP, respectively, and hybridized on glass array 2 X105 K at 65 °C for 40 h at 20 rpm. Slides were then washed and scanned on an Agilent 4000C dual laser scanner, and images were analyzed with Feature Extraction v10.5 software. Raw txt files were then loaded into Cytogenomics software for data processing and visualization.

### Fish analysis

FISH analysis was performed using the following probes: ALK (2p23) Break Apart Rearrangement probe (Vysis, Downers Grove, IL, USA), dual color AURKA (20q13)/CEN20 probe (Kreatech Diagnostics, Amsterdam, Netherlands), dual color EGFR (7p12)/CEP7 probe (Vysis), dual color HER2 (17q11.2)/CEP17 (DAKO, Glostrup, Denmark), dual color MET (7q31.2)/CEP7 probe (Vysis) and dual color TP53 (17p13.1)/CEP17 (Vysis) and Del(5q) Deletion probe (Cytocell, Cambridge, UK). FISH was carried out on interphase cells prepared according to standard techniques. Cells were incubated with the probe for 5 min at 75 °C for co-denaturation and placed in a humidified chamber at 37 °C overnight for the hybridization step. After washing, chromatin was counterstained with DAPI II (Vysis). An average of 100 cells was analyzed using a Olympus BX61 microscope and CytoVision software. *AURKA*, *EGFR*, *HER2*, *MET*, and *TP53* gene *status* was defined evaluating the *ratio* between the gene copy number (CN) and the relative centromere number (*ratio* CN/CEN) and the mean copy number of gene (*ratio* CN/nuclei). A gene amplification was defined when the *ratio* CN/CEN was ≥2.0 and a gene/centromere gain when the mean copy number was ≥3.

### Mutational analysis

Genomic DNA was extracted by using QIAamp DNA FFPE Mini kit (Qiagen, Milan, Italy) following the manufacturer’s instructions. The kinase domain of EGFR coding sequence, from exons 18 to 21, was amplified by using primers and nested polymerase chain reaction (PCR) conditions previously described by Lynch et al. [35]. Exon 2 of K-RAS, exons 9 and 20 of PI3KCA, and exon 15 of B-RAF were amplified by PCR as previously described [36]. PCR products were then purified using Wizard^®^ SV Gel and PCR Clean-Up System (Promega, Italy), and sense and antisense sequences were obtained using forward and reverse internal primers respectively. Each exon was sequenced using the BigDye Terminator Cycle sequence following the PE Applied Biosystem strategy and Applied Biosystem ABI PRISM3100 DNA Sequencer (Applied Biosystem, Forster City, CA). Mutations were confirmed performing two independent rounds of PCR amplifications.

## Results

### Generation and immunophenotyping of ICC cell line

For the cell isolation, tumor tissue derived from the PDX at the fourth generation was mechanically and enzymatically dissociated. Cells adhered to the dish 24 h after plating, but only 1 week later, they formed scattered colonies. After 1 month in the optimal cell culture conditions, Knockout/DMEM/F-12 medium in the presence of 10 % FBS and P/S, cells were detached for the first time. At the 25th passage, after about 5 months, a stable cell line was obtained and named MT-CHC01. It appears as a homogeneous culture of tumor cells with epithelial morphologic features (Supplementary Fig. 1A and B) and is mycoplasma free (Supplementary Fig. 1C).

**Fig. 1:**
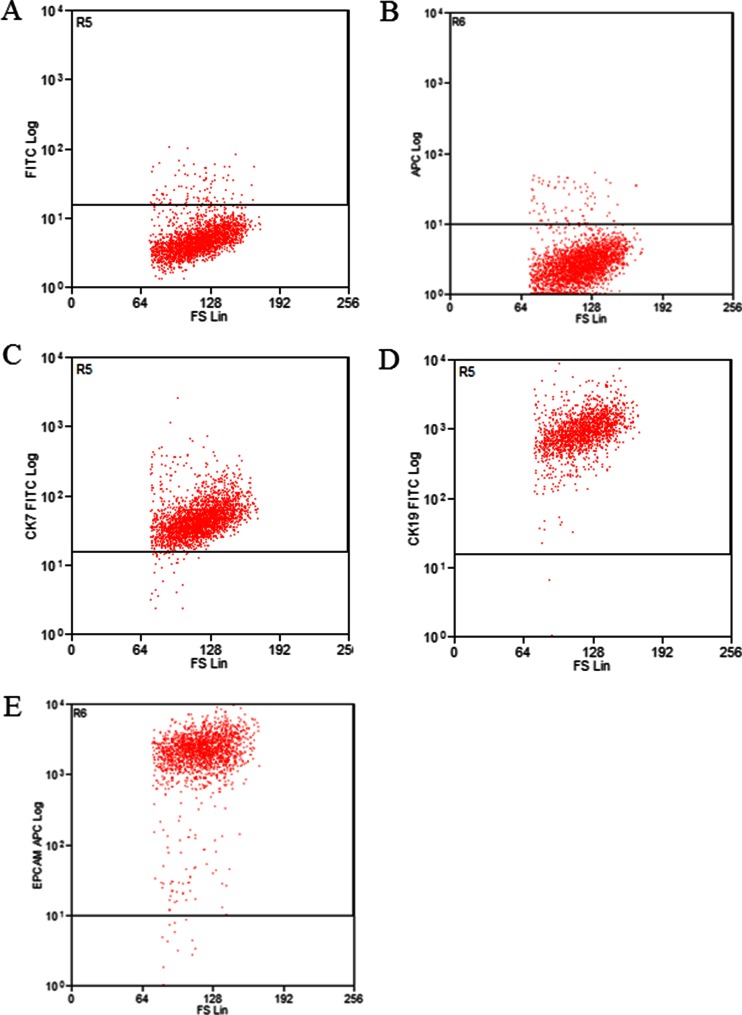
Immunophenotyping of ICC cell line. Epithelial cell markers are expressed in established MT-CHC01 cells. **a**, **b** Negative control: cells incubated with antibody isotype as primary antibody. Panels from C to E represent FACS analysis of CK7 (98.99 %) (**c**), CK19 (98.78 %) (**d**), and EPCAM (99.19 %) (**e**)

Immunophenotypical analysis revealed that MT-CHC01 cell line expressed epithelial cell markers, such as EPCAM (99.19 %), CK7 (98.99 %), and 19 (99.78 %) as shown in Fig. 1 [14, 27].

**Fig. 2:**
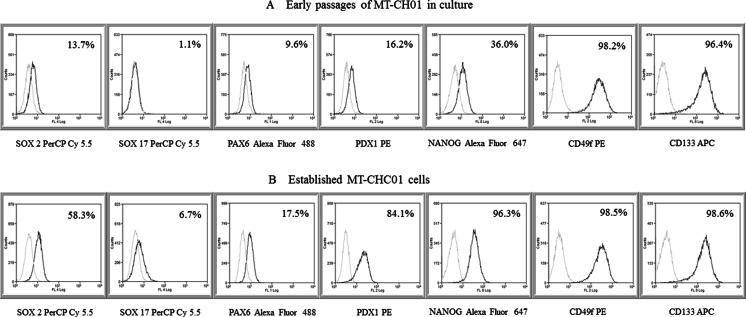
Expression of stemness and pluripotency markers in MT-CHC01 cell line at two different passages. Representative FACS analyses of SOX2/17, PAX6, PDX1, Nanog, CD49f, and CD133 in early primary (**a**) and established (**b**) cell culture

The expression of different stemness and pluripotency markers, already known to be typical of human biliary tree [37] or others characteristics of other tumor types, was also investigated. The established MT-CHC01 cells expressed high levels of SOX2 (58 %), Nanog (96.3 %), CD49f/integrin α6 (98 %), CD24 (78 %), PDX1 (84.1 %), FOXA2 (94.2 %), and CD133 (95.2 %) (Supplementary Table 1). Most of the same markers, analyzed at early passages (Fig. 2a) were not expressed; only CD133 and CD49f were at high levels, while Nanog was expressed in about 30 % of the population versus 96.3 % of established MT-CHC01 cells (Fig. 2b). This suggests that a phenotypic selection occurred in cultured conditions.

### Biological characterization of MT-CHC01 cell line

MT-CHC01 cells were able to grow in a monolayer in appropriate culture medium. After cell line stabilization, the population doubling time was determined. In optimal culture conditions, estimated as 1.4 × 10^5^ cells/cm^2^, the population doubling time was about 40 h (Supplementary Fig. 2).

**Fig. 3:**
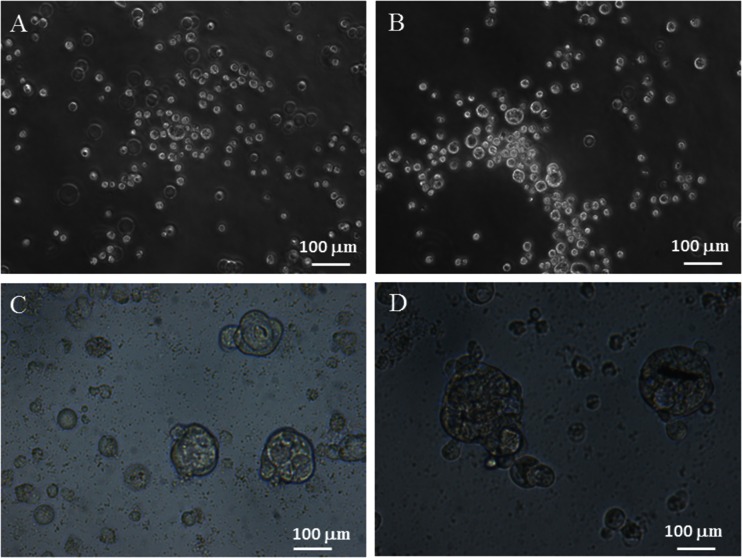
**a**, **b** Representative images of anchorage-independent growth of MT-CHC01 cells in soft agar. Cells were suspended in 0.3 % soft agar layered over 0.3 %. Medium was added twice a week for 3 weeks, and colonies and cell aggregates were microscopically observed after 3 weeks. **c**, **d** Representative images of spheres obtained from MT-CHC01 cells after 14 days of culture. Cells were seeded (ultra-low attachment plate in stem cell medium (SC medium: DMEM-F12 medium, 1× B27, 200 ng/mL human EGF, 10 ng/mL human FGF, 0.4 % BSA, 4 μg/mL insulin and 100 U/mL penicillin/100 μg/mL streptomycin). Sphere formation was monitored on days 7, 10, and 14 after seeding. *Bars* correspond to 100 μm

Plating MT-CHC01 cells on soft agar medium for 3 weeks, they were able to grow in an anchorage-independent manner (Fig. 3a, b).

To confirm the stem cell status emerged from the expression analysis of several stemness and pluripotency markers, a sphere-formation assay was performed. As shown in Fig. 3c, d, MT-CHC01 cells clearly formed spheres in low attachment and serum-free conditions.

**Fig. 4:**
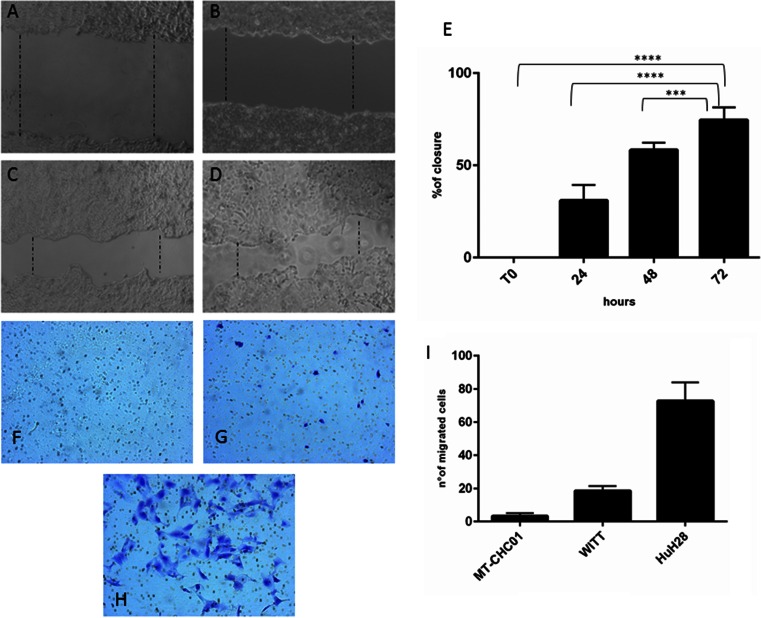
Migration ability of MT-CHC01 cells. **a**–**d** Wound healing assay: MT-CHC01 cells were seeded in six-well tissue culture plates and allowed to grow until 100 % confluence. The cell layer was “wounded” and cell migration toward the scraped area was observed after the wounding (**a**), 24 (**b**), 48 (**c**), and 72 h (**d**) in optimal culture conditions. **e** Bars representing the area of closure at different time points; 100 % corresponds to the area at the time of wounding. **e**–**g** Transwell migration assay on MT-CHC01 (**e**), WITT (**f**), and HuH28 (**g**) cells. Cells were seeded to the upper surface of a transwell chamber. The upper and lower cultures were separated by a 8-μm pore size poly-vinyl-pyrrolidone-free polycarbonate filters. **i** *Bars* represent the number of migrated cells. After 48 h of incubation period, filters were fixed with methanol and stained with 0.5 % crystal violet in 25 % methanol and the cells on the upper surface of the filters were removed using cotton swabs. Cells invading the lower surface were counted in five random fields and expressed as number of invaded cells per well

The MT-CHC01 motility was investigated by using both wound healing and transwell chamber assays. Figure 4a–d shows that the wound in MT-CHC01 cells was significantly closed at 24, 48, and 72 h after the wound; the migration potential of MT-CHC01 is lower if compared to other commercial cell lines such as HuH28 (Fig. 4e), that closes the wound within 24 h as previously demonstrated [38]. This data was confirmed by transwell chamber assay (Fig. 4f–i), in which we observed that MT-CHC01 cells had a lower migration potential rate (Fig. 4f) compared with the WITT (Fig. 4g) and the HuH28 (Fig. 4h) cell lines.

### Tumorigenicity in vivo

To verify if the established cell line retained the tumorigenicity in vivo, NOD/SCID mice were subcutaneously injected with 3.0 × 10^6^ viable cells. In three independent experiments of ten mice each, MT-CHC01 cells were able to develop tumor in all animals. After 1–2 weeks, tumors reached a volume ranging from 245 to 500 mm^3^; a cohort of mice (*n* = 10) was monitored for other 4 weeks when tumors reached volumes ranging from 1500 to 2100 mm^3^ (Supplementary Fig. 3); no evidence of local invasion nor distant metastasis was revealed. Hematoxylin/eosin staining and AFP, CA19-9, and CEA immunohistochemical analyses were also performed on primary and PDX tumors, on MT-CHC01 cells, and on MT-CHC01 xenograft, revealing the same tissue architecture and pathological phenotype of ICC [39] (Supplementary Fig. 4).

### Genetic characterization of MT-CHC01 cell line

#### Mutational analysis

**Fig. 5:**
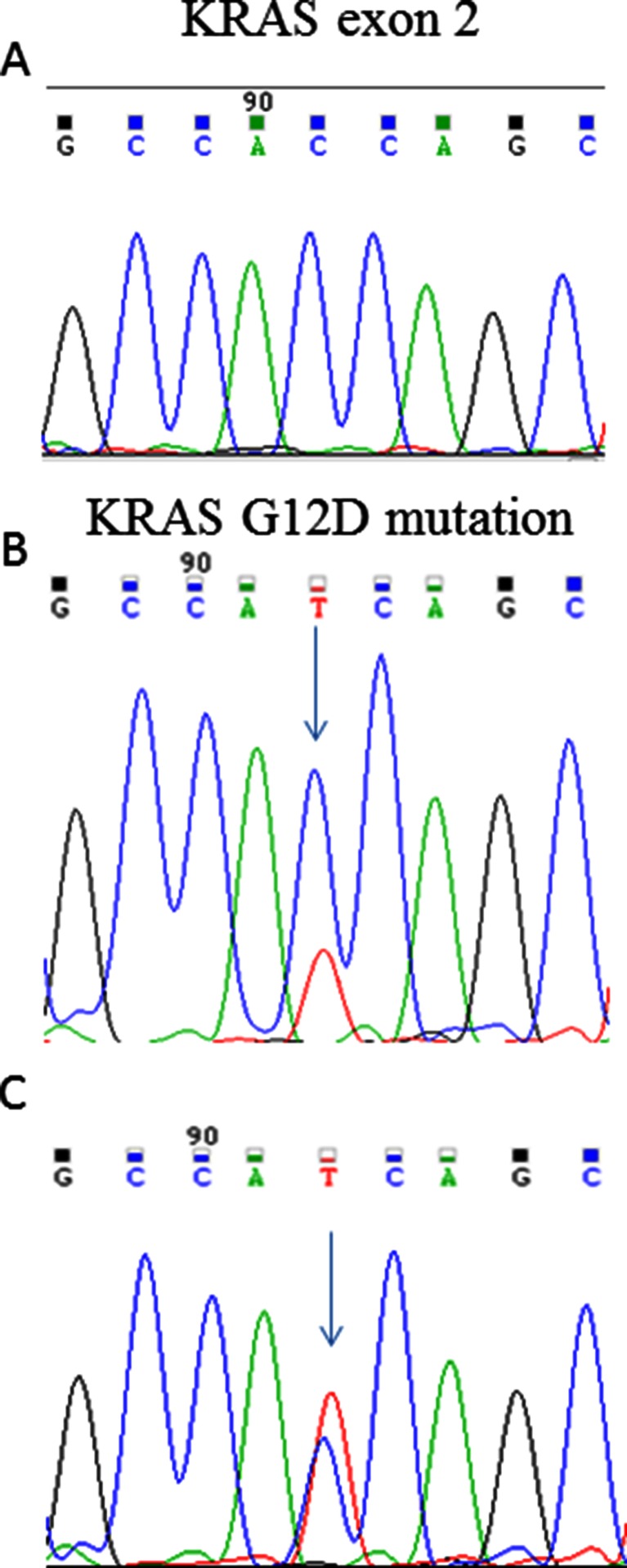
Electropherograms of K-RAS (exon 2). Wild-type sequence of K-RAS exon 2 in WITT cells (**a**), and K-RAS G12D mutation found in primary tumor (**b**) and in MT-CHC01 cell line (**c**)

Mutational analysis of the kinase domain of EGFR coding sequence (exons 18 to 21), exon 2 of K-RAS, exons 9 and 20 of PI3KCA, and exon 15 of B-RAF was performed in primary tumor, and in MT-CHC01 cell line. As shown in Fig. 5, only the sequence of K-RAS exon 2 was mutated (G12D mutation) both in primary (Fig. 5b) and in MT-CHC01 cells (Fig. 5c).

#### Conventional karyotyping and aCGH

**Fig. 6:**
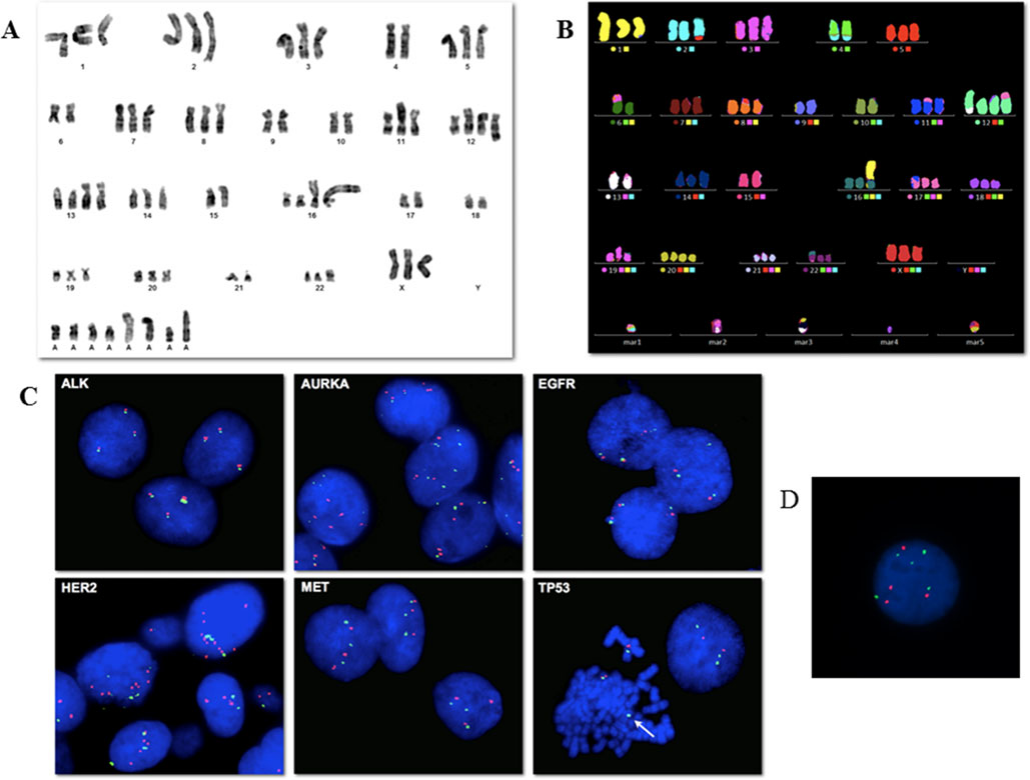
Genetic characterization of MT-CHC01 cells. Representative G-banded karyotype (**a**) and M-FISH karyogram (**b**) of the MT-CHC01 cell line. Note the presence of many marker chromosomes (mar). **c**. FISH analysis of *ALK* (*red*/*green* fusion signal); *AURKA*, *EGFR*, *HER2*, *MET*, and *TP53* genes (*red* signals) and relative centromeres (*green* signals). The *white arrow* shows the *TP53* loss on metaphase. **d**. FISH analysis with a dual color probe specific for 5p15.31 and 5q31.2

To further genetically characterize MT-CHC01 cells, we performed the chromosome G-banding analysis. The cytogenetic analysis on ten metaphases of MT-CHC01 cells demonstrated a highly complex karyotype with a hypotriploid to hypertriploid modal number (3n+/−) (52 to 77 chromosomes). Each chromosome harbored either numerical and/or structural aberrations. M-FISH confirmed that the cell line was near-triploid containing multiple chromosomal aberrations and various marker chromosomes. The composite G-banded and M-FISH karyotype (Fig. 6a, b, respectively), including all clonal chromosomal aberrations observed in ten metaphases of MT-CHC01 cells, was 52~77 < 3n > XXX,-1,t(1;11)(q12;p12-14),der(2)t(2;5)(q31;q21),+3,-4,der(4)t(2;4)(q31;q26)×2,-6,del(6)(q14-16),der(6)t(3;6)(p21;p21),der(8)t(5;8)(p13;p12),-9,-10,der(11)t(11;17)t(p11;q12),der(12)t(12;17)(p12;q12)×2,+der(12)t(12;13)(q24;q14),der(13)t(13;17)(p11;q12),-15,+der(16)t(1;16)(p13;p21),+der(16)t(1;16)(?q21;?p13)ins(16;?)(?p13;?),-17,-18,der(22)t(16;22)(q10;?q10),+6mar[cp10]. Numerical and structural alterations were present in all chromosomes, except chromosomes 9 and 10. In particular, loss of chromosomes 4 and 6, gain of chromosome 16, der(4)t(2;4)(q31;q26), der(8)t(5;8)(p13;p12) were present in all metaphases [10/10], followed by der(2)t(2;5)(q31;q21), del(6)(q14-16), der(22)t(16;22)(q10;?q10) [8/10], der(12)t(12;13)(q24;q14), der(12)t(12;17)(p12;q12) [7/10], t(1;11)(q12;p12-14), der(13)t(13;17)(p11;q12), der(16)t(1;16)(?q21;?p13)ins(16;?)(?p13;?) [6/10]. Chromosomes 1, 5, and 17 were the most frequently involved. Supplementary Fig. 5 showed the chromosome ideogram of a median of all structural aberrations observed in MT-CHC01 cell line. The complex karyotype of MT-CHC01 was further confirmed by comparative genomic hybridization array (aCGH) (Supplementary Fig. 6).

#### FISH analysis on interphase nuclei

Interphase FISH was used to assess the *status* of *ALK* (2p23), *AURKA* (20q13), *EGFR* (7p12), *HER2* (17q11.2), *MET* (7q31.2), and *TP53* (17p13.1) genes with relation to the observed chromosomal aberrations. *ALK*, *EGFR*, and *MET* genes showed a trisomic pattern of signals reflecting the near-triploid chromosomal assessment. As shown in Fig. 6c, a low level of *HER2* gene amplification (*ratio* CN/CEN, 2.1) was observed, consistent with 17q instability and its involvement in various rearrangements with multiple partner chromosomes. Moreover, a loss of *TP53* was present if compared to chromosome 17 centromere (TP53 mean copy number 2.2 vs CEN17 mean copy number 3.1) as a consequence of 17p loss detected in the karyotype. We observed a gain of *AURKA* gene (mean copy number 5.3) and chromosome 20 centromere (mean copy number 4.1) not detectable in the karyotype, as only three copies of chromosome 20 were always present. Finally, to confirm the deletion on chromosome 5 detected by CGH array, we performed a FISH analysis with a dual color probe specific for 5p15.31 and 5q31.2 regions. As expected, a loss of 5q31.2 region was detected if compared to the copy number of 5p15.31 region (5p15.31 mean copy number 5 vs 5q31.2 mean copy number 4) (Fig. 6d).

## Discussion

Biliary tract carcinoma is a complex and heterogeneous disease, characterized by different incidence, etiology, genetic, and molecular profiles. In Italy, BTC shows progressive increase in incidence mainly in intrahepatic cholangiocarcinoma [40].

The importance of identifying preclinical models able to closely reflect the characteristics of tumor of origin is crucial. To date, all models available have limits and consist of cell lines derived from oriental patients. None of these models derived from patient living in Europe and in particular in Italy.

In this work, we characterized a preclinical model represented by a cell line obtained from an Italian intrahepatic cholangiocarcinoma PDX.

The MT-CHC01 cell line was extensively characterized from biological, molecular, and genetic point of view.

The MT-CHC01 cells grew as an adherent monolayer with a population doubling time of approximately 40 h. The morphology was clearly epithelial, and this status was confirmed by immunophenotypic analysis, demonstrating that they expressed epithelial cell markers, EPCAM, CK7, and CK19. In soft agar medium, MT-CHC01 cells revealed the ability to grow in anchorage-independent manner as cellular aggregates; in low attachment and serum-free culture conditions in stem cell medium, MT-CHC01 originated spheroid structures. A peculiar characteristic of cancer cells is their ability to migrate; thus, we investigated the in vitro motility of the MT-CHC01 cells. As demonstrated by wound healing and transwell chamber assays, the MT-CHC01 cells showed a low migration potential compared to other BTC cell lines, reflecting the reduced metastatic potential of this tumor.

Immunophenotyping characterization demonstrated that MT-CHC01 cells expressed, at high levels, some of stemness and pluripotency markers such as SOX2, Nanog, CD49f, CD24, PDX1, FOXA2, and CD133. Their expression was increased during cell stabilization; at early passage of culture, the other tested markers, with the exception of CD133, CD49f, and Nanog, were scarcely represented. This could suggest that during cell propagation, a clone selection with putative cancer stem cell phenotype occurred, as demonstrated by Rowehl et al. in a colorectal cancer cell line model [41]. The ability to grow in suspension, to form spheres, to migrate, and to be tumorigenic if s.c. injected in mice recipient, further supported this hypothesis. Cancer stem cells represent a small population which sustain tumor growth and have the capabilities of self-renewal and multilineage differentiation; they also play an important role in carcinogenesis and in resistance to therapies (chemotherapy and target therapy) [42–44]. Our cell line, which has characteristics of putative stem cells, could be an useful preclinical model to identify specific molecular targets of this subpopulation and evaluate the effectiveness of combination therapies designed to eradicate cancer stem cells and cancer.

It is known that tumor cells progressively acquired many genetic and molecular alterations. Cytogenetic analysis of MT-CHC01 cells confirmed a highly complex karyotype with a hypotriploid to hypertriploid modal number (52 to 77 chromosomes), multiple chromosomal aberrations, and various marker chromosomes. With the exception of chromosomes 9 and 10, numerical and structural alterations were observed, in particular in chromosomes 1, 4 (loss), 5, 6 (loss), 16 (gain), and 17 (loss). Only few data are available in literature on cytogenetic of BTC, but most of them, derived from BTC cell lines karyotyping, showed chromosomal aberration in chromosomes 2, 4, 5, 7, 8, 9, 13, 17, 18, 19, X, and Y [13, 26, 28, 45, 46]. The complex karyotype of MT-CHC01 was further confirmed by aCGH analysis; it revealed a huge number of chromosomic aberrations; in particular, there is the gain of chr 2q, 3q, 12p, and the loss of 3p, 5q, 6p, 8p, 9p, 18q, already described in BTC by Rijken and collaborators [45, 47].

Further, genetic characterization was focused on mutational status of the hotspot of EGFR (from exons 18 to 21) and its principle signal transducers (exon 2 of K-RAS, exons 9 and 20 of PI3KCA, exon 15 of B-RAF); only a mutation of KRAS (G12D) was found. Other genomic analyses revealed loss of TP53, *ALK*, *EGFR*, and *MET* genes showed a trisomic pattern of signals, low level of *HER2* gene amplification, and a gain of *AURKA* gene.

In conclusion, we established a human intrahepatic cholangiocarcinoma cell line, derived from an Italian patient, which could represent a useful tool to better characterize this disease in relation to etiology and ethnicity and to develop new therapies for ICC patients.

## Electronic supplementary material

Below is the link to the electronic supplementary material.

ESM 1 (DOCX 2335 kb)
