# Supplementary material for: Establishment and characterization of a human intrahepatic cholangiocarcinoma cell line derived from an Italian patient
Source: Tumour Biol. 2015 Oct 20;37(3):4041–52. doi: 10.1007/s13277-015-4215-3 (PMC4844644; doi:10.1007/s13277-015-4215-3)
Supplement: Supplementary file 1 — (DOCX 2335 kb) [file 13277_2015_4215_MOESM1_ESM.docx]

**Title: Establishment and characterization of a human intrahepatic cholangiocarcinoma cell line derived from an Italian patient**

**Giuliana Cavalloni^1,§^, Caterina Peraldo Neia^1§^ , Chiara Varamo^2^, Laura Casorzo^3^, Carmine Dell’Aglio^3^, Paola Bernabei^4^, Giovanna Chiorino^5^ , Massimo Aglietta^1,2^, Francesco Leone^1,2^**

^1^ Medical Oncology Division, Fondazione del Piemonte per l’Oncologia (FPO), Candiolo Cancer Institute IRCCS, Italy;

^2^ University of Turin; Department of Oncology, Candiolo Cancer Institute IRCCS, Italy;

^3^ Unit of Pathology FPO, Candiolo Cancer Institute IRCCS, Italy;

^4^ Flow Cytometry Center, (FPO), Candiolo Cancer Institute IRCCS, Italy.

^5^ Cancer Genomics Laboratory, Fondazione Edo ed Elvo Tempia Valenta, Biella, Italy

**^§^** The first two authors contributed equally to this work

**Corresponding Author: Giuliana Cavalloni:** [giuliana.cavalloni@ircc.it](mailto:giuliana.cavalloni@ircc.it)

IRCCS-Institute Candiolo, Strada Provinciale 142, Km 3,95, 10060 Candiolo, (Turin), Italy

Phone Number: +390119933503. Fax Number: +390119933299

**Caterina Peraldo Neia:** [caterina.peraldoneia@ircc.it](mailto:caterina.peraldoneia@ircc.it)

IRCCS-Institute Candiolo, Strada Provinciale 142, Km 3,95, 10060 Candiolo, (Turin), Italy;

**Chiara Varamo:** [chiara.varamo@ircc.it](mailto:chiara.varamo@ircc.itI) IRCCS-Institute Candiolo, Strada Provinciale 142, Km 3,95, 10060 Candiolo, (Turin), Italy

**Laura Casorzo**: [laura.casorzo@ircc.it](mailto:laura.casorzo@ircc.it)

IRCCS-Institute Candiolo, Strada Provinciale 142, Km 3,95, 10060 Candiolo, (Turin), Italy

**Carmine Dell’Aglio:** [carmine.dellaglio@ircc.it](mailto:carmine.dellaglio@ircc.it)

IRCCS-Institute Candiolo, Strada Provinciale 142, Km 3,95, 10060 Candiolo, (Turin), Italy

**Paola Bernabei:** paola.bernabei@ircc.it

IRCCS-Institute Candiolo, Strada Provinciale 142, Km 3,95, 10060 Candiolo, (Turin), Italy

**Giovanna Chiorino:** [giovanna.chiorino@gmail.com](mailto:giovanna.chiorino@gmail.com)

Fondazione Edo ed Elvo Tempia Valenta, Via Malta 3, 13900, Biella Italy

**Massimo Aglietta:** [massimo.aglietta@ircc.it](mailto:massimo.aglietta@ircc.it)

IRCCS-Institute Candiolo, Strada Provinciale 142, Km 3,95, 10060 Candiolo, (Turin), Italy

**Francesco Leone:** [francesco.leone@ircc.it](mailto:francesco.leone@ircc.it)

IRCCS-Institute Candiolo, Strada Provinciale 142, Km 3,95, 10060 Candiolo, (Turin), Italy;

**Supplementary table 1**. Representative phenotypic profiling of MT-CHC01 cell line by FACS analysis

| Marker | % of positive cells |
| --- | --- |
| SOX2 | 58 |
| SOX17 | 6.7 |
| OCT3/4 | 0.3 |
| NANOG | 96.3 |
| PAX6 | 17.5 |
| c-KIT/CD117 | 3.7 |
| CD34 | 2.5 |
| KDR/VEGFR-2 | 9.6 |
| Integrin α6/CD49f | 98.1 |
| CD24 | 78.3 |
| CD44 | 36.2 |
| CXCR4 | 5.7 |
| PDX1 | 84.1 |
| STRO1 | 8.8 |
| FOXA2 | 94.9 |
| CD133 | 95.2 |
| ABCG2/CD338 | 0.1 |

**Supplementary figure legends**

**Supplementary figure 1. Panel A and-B morphology of MT-CHC01 in culture.** The cells grow in monolayer and exhibit epithelial morphology(A 10X magnification, B 20X magnification).

**Panel C. PCR amplification for mycoplasma contamination detection.** Lane 1: 100 bp DNA ladder; lane 2: MT-CHC01 mycoplasma negative sample (191 bp); lane 3: the 16S rRNA coding region of mycoplasma genome (267 bp).

**Supplementary figure 2. Growth curve of MT-CHC01 cells:** 1.4x10^5^ cells were plated in 24-well plates in triplicate in three different experiments in optimal medium. Viable cells were counted at 24, 48 and 72 hours after seeding.

**Supplementary figure 3. Tumor formation in NOD/SCID mice injected with MT-CHC01 cells. Panel A**. Representative engraftments in two mice after s.c. injection of 3.0x10^6^ MT-CHC01 cells. **Panel B.** Two representative explanted tumors generated from MT-CHC01 cells. **Panel C.** Curve of the in vivo growth of MT-CHC01.

**Supplementary figure 4:** Immunohistochemistry analysis for the expression of AFP, CA19-9, CEA, and H/E staining of primary tumor, PDX, MT-CHC01 cell line and its xenograft. 20X Magnification.

**Supplementary figure 5. Chromosome ideogram of all structural aberrations in MT-CHC01 cell line.** Characters above ideograms indicate the derivative chromosomes recognized with conventional cytogenetic analysis and M-FISH. (A/H) t(1;11)(q12;p12-14); (B) der(2)t(2;5)(q31;q21); (C) der(4)t(2;4)(q31;q26); (D) del(6)(q14-16); (E) der(6)t(3;6)(p21;p21); (F) der(8)t(5;8)(p13;p12); (G) der(11)t(11;17)(p11;q12); (I) der(12)t(12;17)(p12;q12); (J) der(12)t(12;13)(q24;q14); (K) der(13)t(13;17)(p11;q12); (L) der(16)t(1;16)(p13;p12); (M) der(16)t(1;16)(?q21;?p13)ins(16;?)(?p13;?), in black the unknown inserted chromosomal segment; (N) der(22)t(16;22)(q11;?p11).

**Supplementary figure 6.** Graphical representation of the genetic aberrations in MT-CHC01 cells by aCGH.

**Supplementary figure 1**


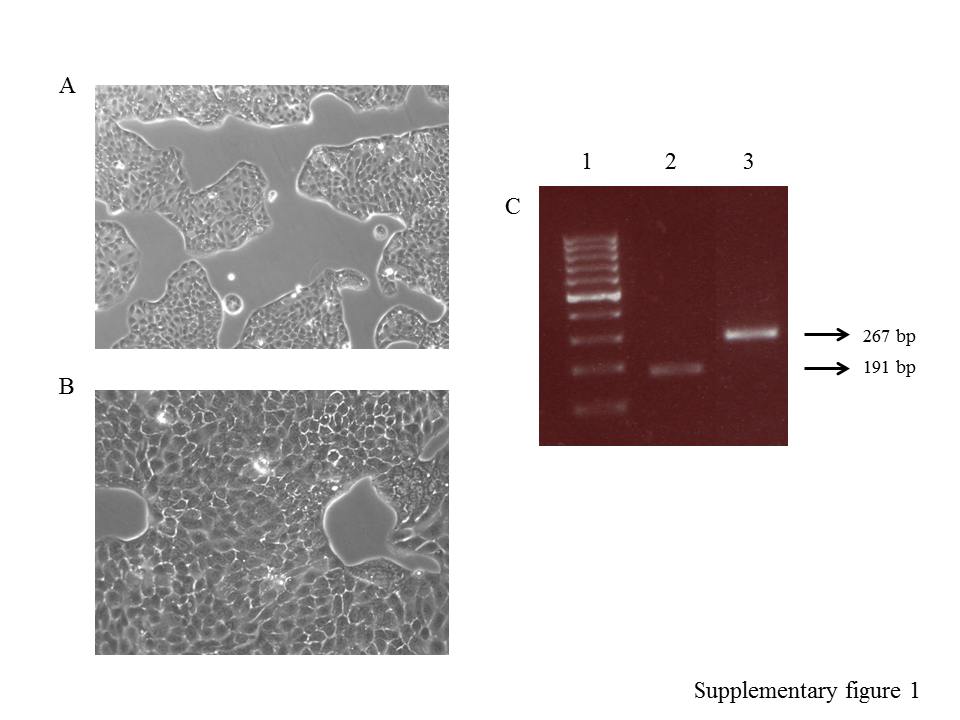


**Supplementary figure 2**


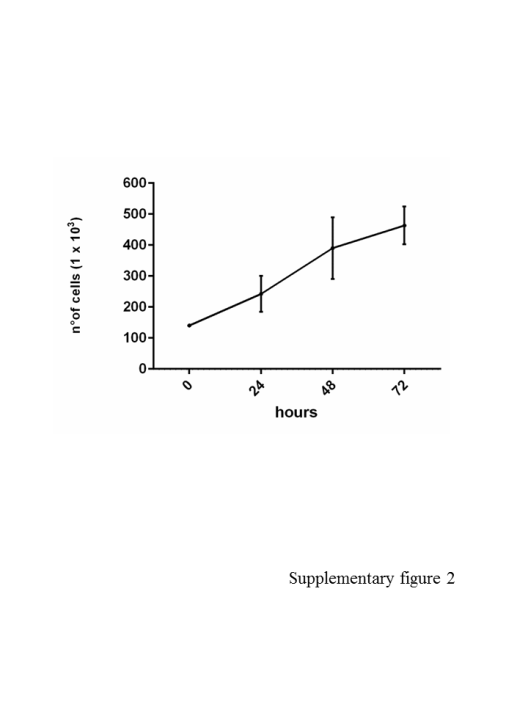


**Supplementary figure 3**


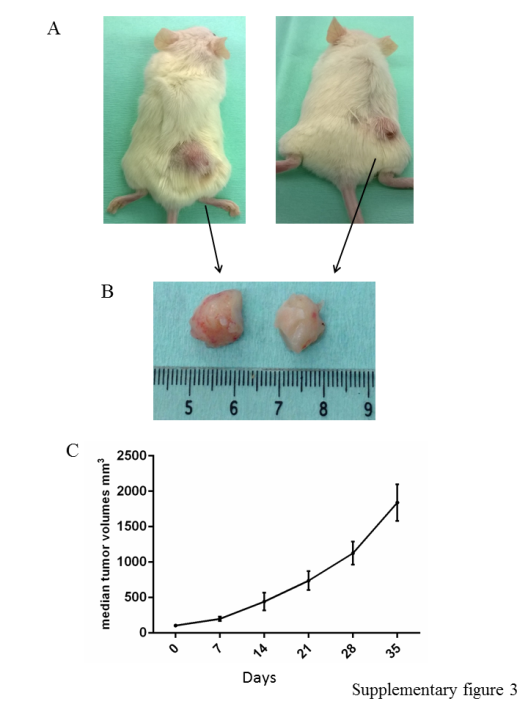


**Supplementary figure 4**

**
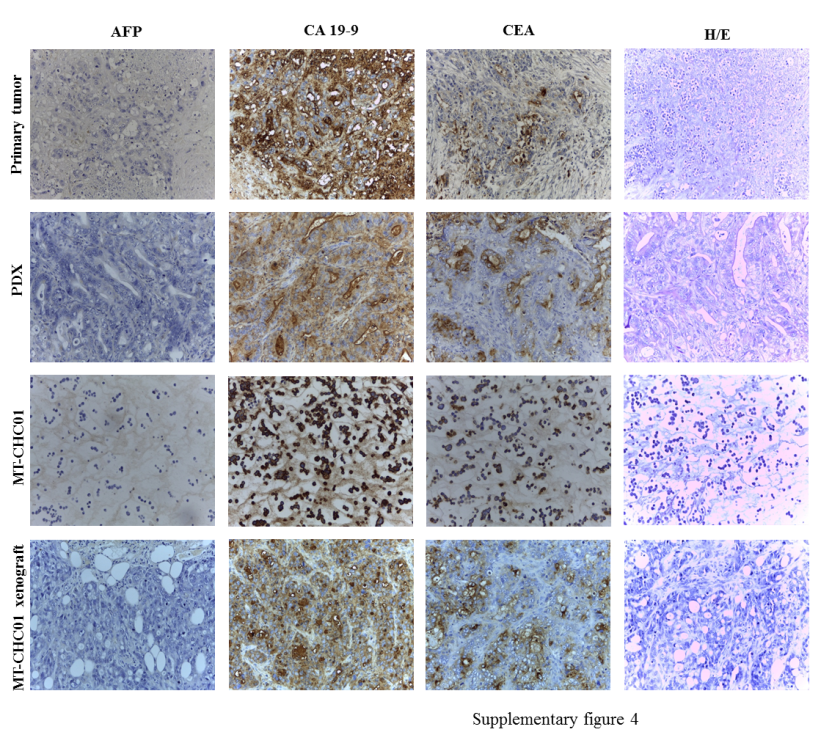
**

**Supplementary figure 5**

**
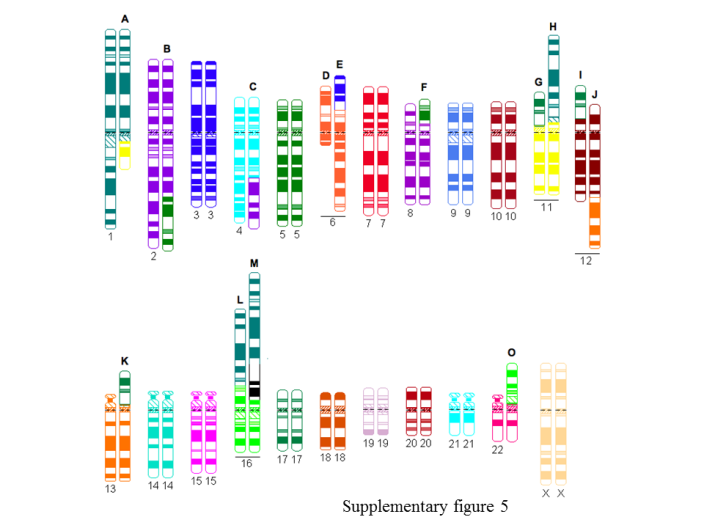
**

**Supplementary figure 6**

**
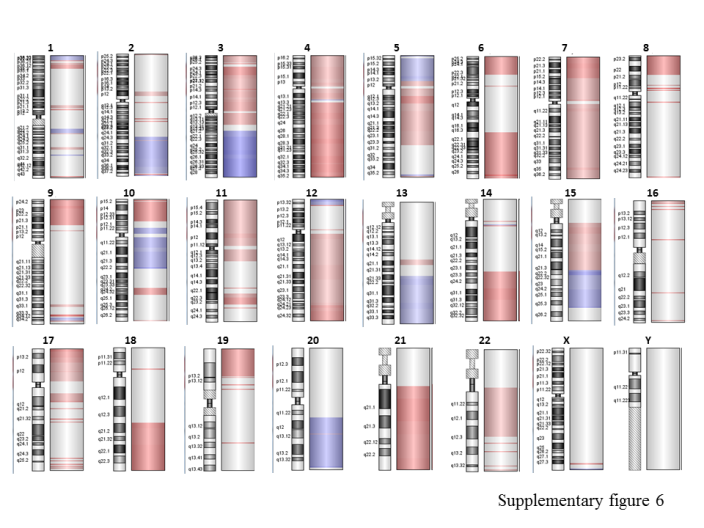
**
